# Accelerated aging induced by deficiency of Zmpste24 protects old mice to develop bleomycin-induced pulmonary fibrosis

**DOI:** 10.18632/aging.101679

**Published:** 2018-12-10

**Authors:** Jazmín Calyeca, Yalbi I. Balderas-Martínez, Raúl Olmos, Rogelio Jasso, Vilma Maldonado, Quetzali Rivera, Moisés Selman, Annie Pardo

**Affiliations:** 1 Facultad de Ciencias, Universidad Nacional Autonoma de México, Mexico City, Mexico; 2 Cátedra Consejo Nacional de Ciencia y Tecnologia (CONACyT)-INER, Mexico City, Mexico; 3 Instituto Nacional de Enfermedades Respiratorias Ismael Cosío Villegas, Mexico City, Mexico; 4 Instituto Nacional de Medicina Genómica, Mexico City, Mexico

**Keywords:** pulmonary fibrosis, aging, microRNAs

## Abstract

Idiopathic pulmonary fibrosis is a devastating aging-associated disease of unknown etiology. Despite that aging is a major risk factor, the mechanisms linking aging with this disease are uncertain, and experimental models to explore them in lung fibrosis are scanty. We examined the fibrotic response to bleomycin-induced lung injury in *Zmpste24-deficient* mice, which exhibit nuclear lamina defects developing accelerated aging. We found that young WT and *Zmpste24(-/-)* mice developed a similar fibrotic response to bleomycin. Unexpectedly, while old WT mice developed severe lung fibrosis, accelerated aged *Zmpste24-/-* mice were protected showing scant lung damage. To investigate possible mechanisms associated with this resistance to fibrosis, we compared the transcriptome signature of the lungs and found that *Zmpste24*(-/-) mice showed downregulation of several core and associated matrisome genes compared with WT mice. Interestingly, some microRNAs that target extracellular matrix molecules such as miR23a, miR27a, miR29a, miR29b-1, miR145a, and miR491 were dysregulated resulting in downregulation of profibrotic pathways such as TGF-β/SMAD3/NF-κB and Wnt3a/β-catenin signaling axis. These results indicate that the absence of *Zmpste24* in aging mice results in impaired lung fibrotic response after injury, which is likely associated to the dysregulation of fibrosis-related miRNAs.

## Introduction

Idiopathic pulmonary fibrosis (IPF) is a progressive and lethal lung disease of unknown etiology and limited therapeutic options [1–3]. Although the pathogenic mechanisms are uncertain, most evidence indicates that IPF results from the hyperactivation of the respiratory epithelium, which secretes a variety of mediators that induce the migration, proliferation, and activation of fibroblasts with the subsequent destruction of the lung parenchyma [2–5]. The median age at diagnosis is ~65 years, and the incidence increases markedly with age, supporting the notion that aging is a driving force for the development of the disease [1–3].

Aging is characterized by exhaustion of stem cell reservoirs, alterations in proteostasis, increased accumulation of damaged DNA, telomere shortening and mitochondrial dysfunction [6]. Excessive dysfunction of most of these aging-associated mechanisms has been identified in IPF [6,7].

However, most biomedical research regarding the pathogenesis of IPF has been performed in mice 6-8 weeks old and studies on naturally aged, wild-type mice are scanty [8]. The main problem lies in the significant practical difficulties associated with the generation of aged mice, including time and cost. The few studies assessing experimental models in older mice have revealed that aging increases the fibrotic lung response to bleomycin-instillation or herpes virus damage [9,10]. However, in another study, it was found that the severity of fibrosis was not different between young and old mice although aged mice demonstrated an impaired capacity to resolve fibrosis [11].

There is growing interest to identify experimental models of accelerated aging. In this context, the senescence-accelerated mouse (SAM), or the genetically modified telomerase deficient mice have been used to study the effect of aging on the development of pulmonary fibrosis [12,13].

Recently, it was shown that *Zmpste24* deficient mice displayed accelerated aging. Zmpste24 is a zinc metalloproteinase responsible for the final cleavage step of nuclear envelop prelamin A, a critical step in its maturation process. Accumulation of immature lamin A is highly toxic to the cell and produce DNA damage, chromatin remodeling and early senescence [14].

*Zmpste24*-deficient mice appear healthy until ~4 weeks, and are indistinguishable from their heterozygous or wildtype littermates. After eight weeks of age, homozygous null mice progressively show a premature aging phenotype including severe growth retardation, hair loss, osteoporosis, dilated cardiomyopathy, muscular dystrophy, lipodystrophy, and their lifespan is shortened to 4-6 months [14,15].

To clarify the role of aging in the development of lung fibrosis and unveiling whether *Zmpste24* deficient mice could be an appropriate aging-model for this purpose, we examined their fibrotic response to bleomycin-induced lung damage.

Unexpectedly, we found that old but not young *Zmpste24* deficient mice seem to be protected to the development of bleomycin-induced lung fibrosis finding that was related to an upregulation of the microRNAs miR23a, miR27a, miR29a, and miR145a. These findings suggest that accelerated aging induced by the absence of *Zmpste24*, result in attenuated fibrotic response and the overexpression of miRNAs that target extracellular matrix related mRNAs.

## RESULTS

### Baseline lung transcriptome in physiological and accelerated aging

**Figure 1:**
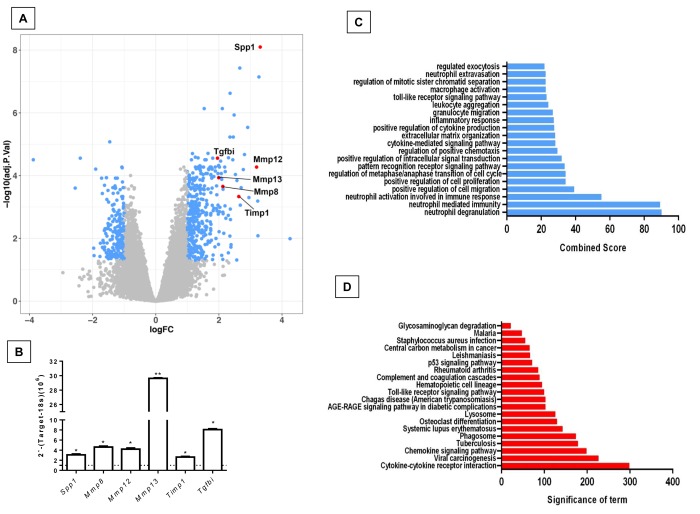
**Differentially expressed genes and pathways in lungs from old compared to young C57/BL6 wild-type mice.** (**A**) Volcano plot of the global gene expression profiling in lungs from old wild type versus young WT mice. Each point represents the difference in expression (log fold-change) between the two groups of mice plotted against the level of statistical significance. Right blue dots represent overexpressed genes, while the left blue dots represent downregulated genes at a significant level of *p< 0.05 and ** p< 0.01. Some Core or associated genes to the Matrisome are plotted in red. (**B**) The expression of these genes was evaluated by quantitative RT-PCR. Bars represent fold-change of old mice over young ones (dotted line). (**C** and **D**) Gene ontology (**C**) and KEGG (**D**) functional analysis of dysregulated genes in old WT mice compared to young littermates. The most significant 20 terms are shown in this figure. Threshold criteria considered for the analysis are log Fold change > 1 or <-1 and p-value < 0.05. KEGG: Kyoto Encyclopedia of Genes and Genomes.

In order to know if there are differences in gene expression between the physiological and accelerated aging process induced by *Zmpste24* deficiency, we performed a global gene expression analysis in lungs from young (4 weeks) and old (82 weeks) WT, and young (4 weeks) and old (16 weeks) *Zmpste24* deficient mice [14]. As shown in the volcano plots, 576 genes were differentially expressed in old WT compared with their young counterpart (Figure 1A). The top 50 upregulated and downregulated genes are presented in Supplementary Table 1. The highest over-expressed gene in old WT lungs compared with young ones was the core Matrisome secreted phosphoprotein-1 (*Spp1* also known as osteopontin). *Tenascin C* and transforming growth factor beta-induced (*Tgf-βi*) genes also belonging to the core Matrisome were highly upregulated. In addition, several matrix metalloproteinases considered as Matrisome-associated genes such as *Mmp8, Mmp12, Mmp13*, and *Timp1* were also upregulated. Some of these findings were validated by qPCR as shown in Figure 1B.

Gene ontology (GO) functional enrichment analysis and KEGG pathways were used for functional analysis of the differentially expressed genes in old and young WT mice. As shown in Figure 1C and D, several pathways were altered in old mice including inflammatory response, positive regulation of cell proliferation and migration and extracellular matrix organization.

**Figure 2:**
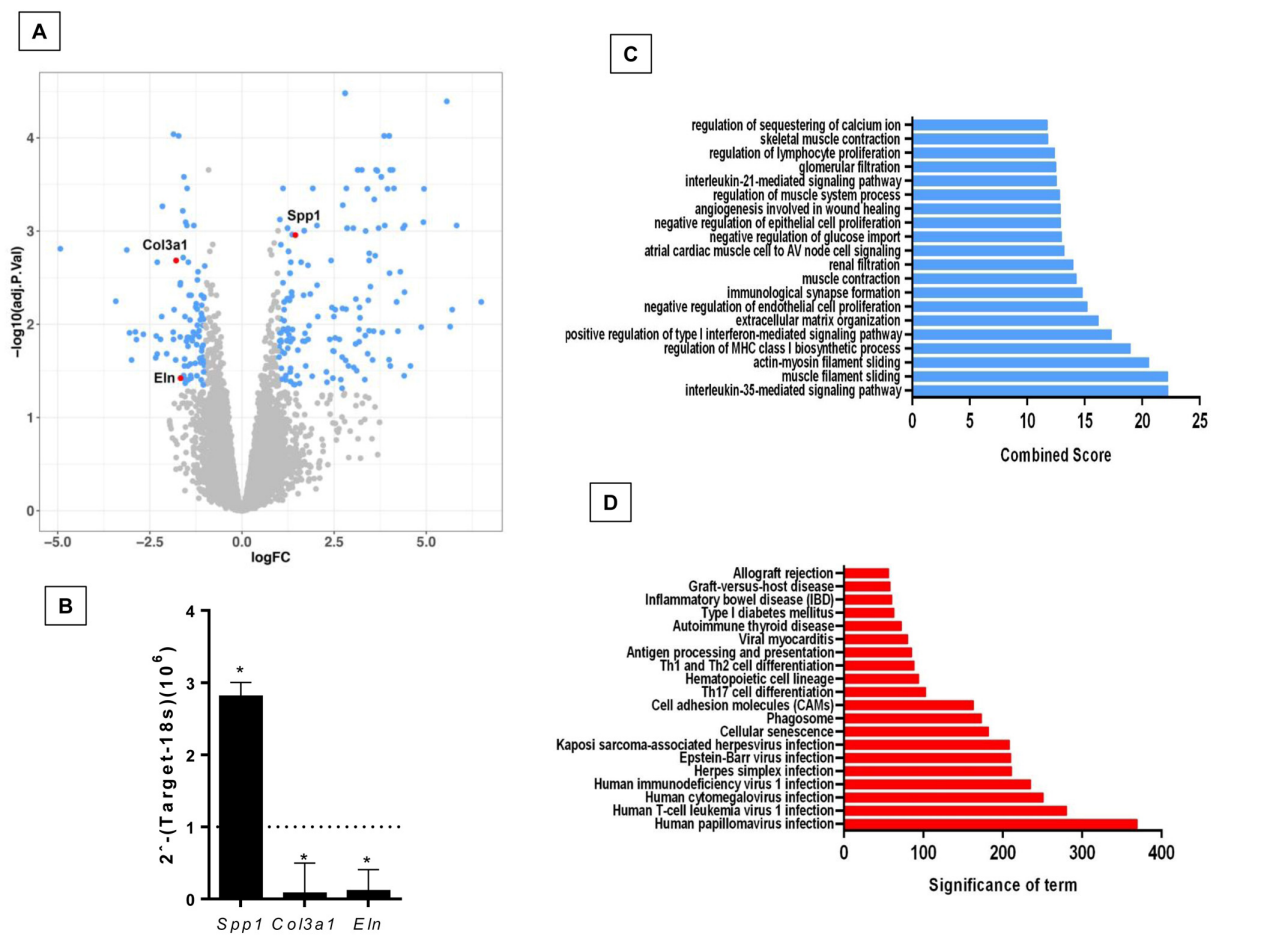
**Differentially expressed genes and pathways in lungs from old compared to young *Zmpste24* deficient mice.** (**A**) Volcano plot of the global gene expression profiling in lungs from old Zmpste24 deficient mice vs young littermates. Each point represents the difference in expression (log fold-change) between the two groups of mice plotted against the level of statistical significance. Right blue dots represent overexpressed genes; left blue dots represent downregulated genes at a significant level of p< 0.05. Some Core Matrisome genes are plotted in red. (**B**) The expression of these genes was evaluated by quantitative RT-PCR. Data are shown as fold-change values as compared with young -/- (dotted lines). (**C** and **D**) Gene ontology (**C**) and KEGG (**D**) functional analysis of dysregulated genes in old Zmpste24 deficient mice compared to young littermates. The most significant 20 terms are shown in this figure. Threshold criteria considered for the analysis are log Fold change > 1 or <-1 and p-value < 0.05.

The comparison of lungs from young and old *Zmpste24* deficient mice revealed 224 differentially expressed genes (Figure 2A). The top 50 upregulated and downregulated genes are shown in Supplementary Table 2. Several genes involved in the immune and inflammatory response, such as chemokines, chemokine receptors, and T-cell receptors were upregulated. As in WT old lungs, *Spp1* was increased. Among the downregulated genes, we found several core Matrisome genes such as elastin (*Eln),* and collagen type 3 alpha-1 (*Col3a1*). These findings were confirmed by qPCR (Figure 2B)*.* Functional analysis by GO and KEGG revealed pathways related to extracellular matrix organization, cell senescence, immune response, as well as several negative regulation pathways such as epithelia and endothelial cell proliferation (Figure 2C and 2D).

Then, when we compared dysregulated genes in natural aging (old versus young WT) with accelerated aging (old versus young *Zmpste24*-deficient) we found 19 overlapping genes between both groups. (Supplementary Figure 1A and Supplementary Table 3). Gene Ontology analyses revealed altered pathways related to immune response, activation of signaling protein activity involved in unfolded protein response among others. (Supplementary Figure 1B and 1C).

### Aging increases lung collagen content in WT and Zmpste24^-/-^ mice and localizes surrounding airways

To evaluate the effect of both physiological and accelerated aging process into lung collagen concentration, we measured hydroxyproline content in the right lungs from WT and *Zmpste24* deficient mice. It is important to emphasize that there were no differences in total weight (13.3 ± 3.5 g vs 12.5 ± 3.4 g) or lung weight (5.5 ± 1.5 mg vs 6.1 ± 1.5mg) between young WT and young *Zmpste24* deficient mice. By contrast, a significant decrease of body and lung weight was observed in old *Zmpste24* deficient mice compared with the WT mice: (body: 14.3 ± 2 g vs 31.5 ± 1.8 g; lung: 8.1 ± 1.6 mg vs 14.1 ± 3.6 mg, *p* ≤ 0.05). For this reason, hydroxyproline was expressed as μg per mg of the dried lung.

**Figure 3:**
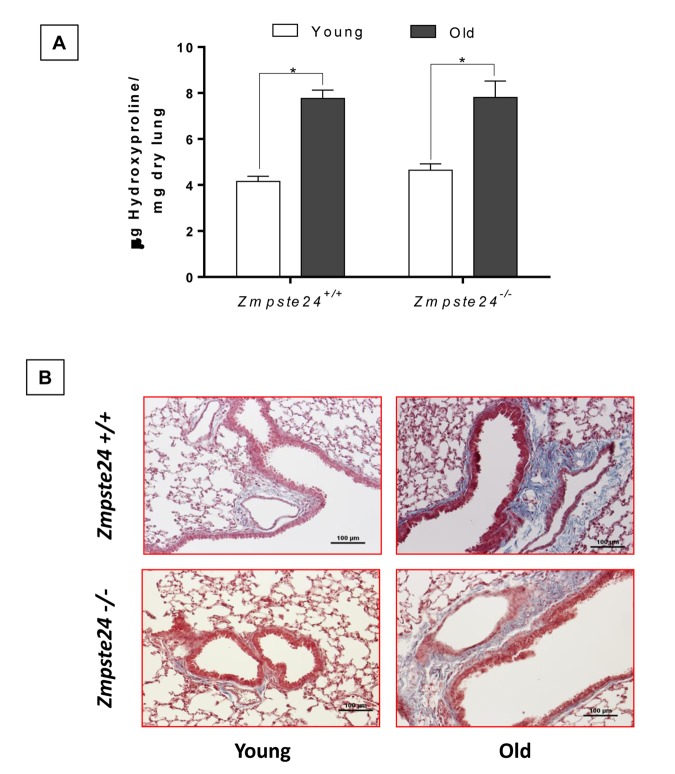
**Aging increases lung collagen content in WT and *Zmpste24*^-/-^ mice.** (**A**) OH-Proline content. Data represent mean ± SD (n=4); *p< 0.05. (**B**) Representative morphological images of two WT and two *Zmpste24* deficient mice stained with Masson’s trichrome. Scale bar, 100 μm.

As illustrated in Figure 3A, lungs from old *Zmpste24* deficient and WT mice showed a significant increase in hydroxyproline content when compared to their respective young littermates (old *Zmpste24* deficient mice: 6.9 ± 1.5 μg/mg vs young 4.2 ± 0.5 μg/mg, and old WT: 6.4 ± 0.6 μg/mg vs young 4.5 ± 0.5 μg/mg, respectively; *p* ≤ 0.05). Interestingly, morphological analysis using Masson’s trichrome staining showed that collagen fibers accumulation occurred mainly surrounding the airways (Figure 3B).

### Young WT and Zmpste24 deficient mice develop similar fibrotic response after bleomycin injury

**Figure 4:**
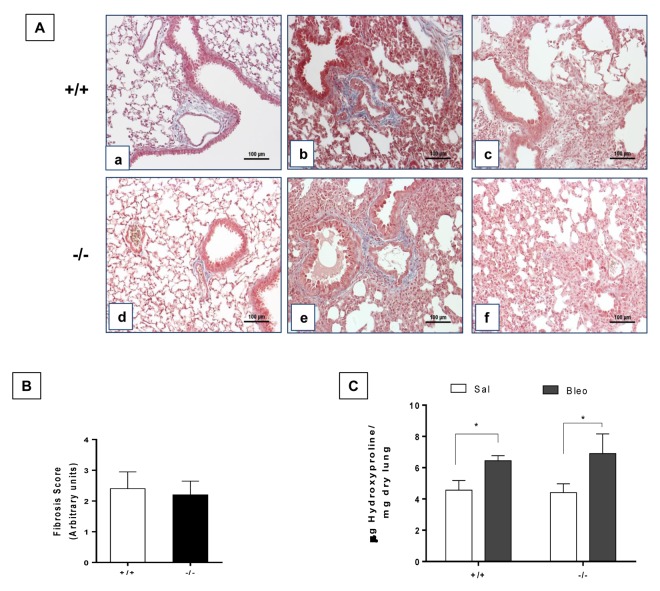
**Young WT and *Zmpste24* deficient mice develop similar fibrotic response after bleomycin injury.** (**A**) Representative Masson Trichrome staining of lung sections from young saline control WT (panel a), and 21 days after bleomycin (panels b, c), and *Zmpste24* deficient mice saline control (panel d) and at 21 d after bleomycin (panels e, f). Scale bar, 100 μm. (**B**) Fibrosis score for grading lung histopathological changes. Graphs represent means ± SD. (**C**) OH-Proline content in lungs after saline or 21 days of bleomycin injury. *p< 0.05; (n=6).

To explore the effect of lung damage in young WT and *Zmpste24* deficient mice, we administered them oropharyngeal bleomycin, and animals were sacrificed 21 days after injury. As illustrated in Figure 4A, both, WT and *Zmpste24* deficient mice displayed similar fibrotic morphological lesions and collagen deposition that was confirmed by the fibrotic score (Figure 4B). OH-proline analysis confirmed this observation showing an increase of lung collagen content after bleomycin without significant differences between WT and *Zmpste24* deficient mice (6.1 ± 0.6 μg/mg, and 6.3 ± 1.3 μg/mg, respectively) (Figure 4C).

### Aging protects Zmpste24 deficient mice from bleomycin-induced pulmonary fibrosis

**Figure 5:**
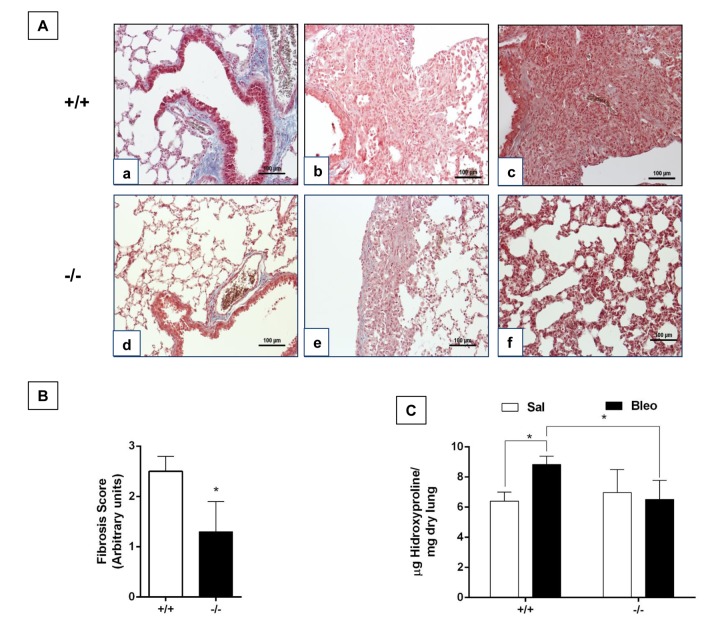
**Aging protects *Zmpste24* deficient mice from bleomycin-induced pulmonary fibrosis.** (**A**) Representative Masson Trichrome staining of lung sections from old saline control WT (panel a), and 21 days after bleomycin (panels b, c), and *Zmpste24* deficient mice saline control (panel d) and at 21 d after bleomycin (panels e, f). Scale bar, 100 μm. (**B**) Fibrosis score for grading lung histopathological changes. Graphs represent means ± SD. *p< 0.05. (**C**) OH-Proline content in lungs after saline or 21 days of bleomycin injury. *p< 0.05; Old WT (n=3); *Zmpste24* deficient mice (n=6).

There is some evidence suggesting that old mice show a more severe lung remodeling after injury compared with young ones [9,10]. In this context, we evaluated the fibrotic response after 21 days of bleomycin administration in old WT mice (82 weeks) and accelerated aging *Zmpste24* deficient mice (16 weeks). Morphological analysis by Masson´s trichrome staining of old WT mice showed severe lung damage and collagen fiber deposition while lungs from old *Zmpste24* deficient mice showed scant damage (Figure 5A). Supporting these data, the fibrotic score was significantly lower in *Zmpste24* deficient mice compared with WT mice (-/- 1.3 ± 0.6 versus WT: 2.5 ± 0.3 p ≤ 0.05) (Figure 5B). Quantification of hydroxyproline content revealed the expected significant increase after bleomycin in old WT mice (8.8 ± 0.5 μg/mg vs saline: 6.4 ± 0.6 μg/mg, p ≤ 0.05). In sharp contrast, lungs from *Zmpste24* deficient mice showed no increase in hydroxyproline content after bleomycin injury (6.5 ± 1.3 μg/mg vs 6.9 ± 1.5μg/mg) (Figure 5C). Taking together, these results suggest that both aging and the absence of *Zmpste24* protect mice for developing pulmonary fibrosis.

**Figure 6:**
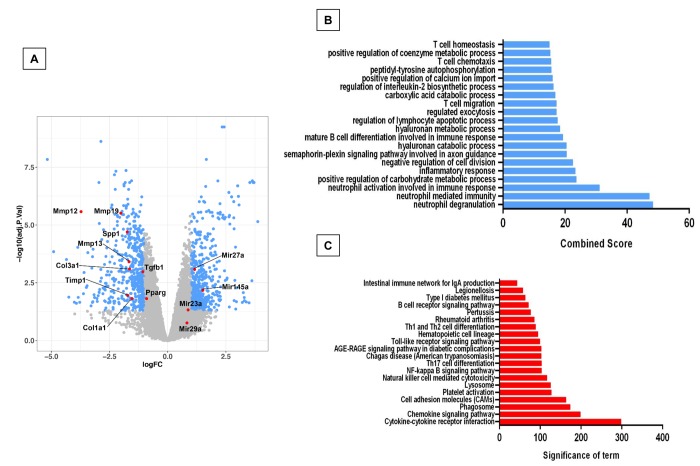
**Microarray analysis of bleomycin-injured lungs of old Zmpste24 deficient mice (n=3) compared with old wildtype littermates (n=3).** Lungs were obtained 21 days after bleomycin injury. (**A**) Volcano plot of the global gene expression profiling in lungs from old injured Zmpste24 deficient mice vs old WT mice. Each point represents the difference in expression (log fold-change) between the two groups of mice plotted against the level of statistical significance. Right blue dots represent overexpressed genes; left blue dots represent relatively downregulated genes at a significant level of p< 0.05. (**B** and **C**) Gene ontology (**B**) and KEGG (**C**) functional analysis. The most significant 20 terms are shown. Threshold criteria considered for the analysis are log fold-change > 1 or <-1 and p-value < 0.05 for genes, and >0.5 or z-0.5 for miRnas.

In order to investigate possible mechanisms that may explain the resistance of the accelerated aged *Zmpste24* deficient mice to bleomycin-induced lung fibrosis, we compared the lung transcriptome signature of the injured old *Zmpste24* deficient mice with those of old WT mice. Whole-transcript array showed 1165 differentially expressed RNAs (Figure 6). Our results revealed that several associated-Matrisome genes including *Mmp12, Mmp13, Mmp19*, and *Timp1* as well as the core Matrisome *Col1a1, Col3a,* *Spp1,* and *Fn1* were downregulated in the old *Zmpste24* deficient mice (Supplementary Table 4). Likewise, some genes with putative antifibrotic effects such as NADPH oxidase 4, transforming growth factor beta receptor III, and decorin were upregulated.

Using key Pathway Advisor-Clarivate we found six transcription factors upregulated including paternally expressed 3 (Peg3), the negative regulator of the inflammatory response nuclear receptor subfamily 4 group A member 1 (NR4A1) RAR-related orphan receptor C (RORC), Krupple-like factor 15 (KLF15), hypoxia-inducible factor 3a, and early growth factor response 1 (Egr1).

GO functional enrichment analysis and KEGG revealed several altered pathways primarily related to an immune/inflammatory response (Figure 6B and C).

### Fibrotic injury induces the expression of antifibrotic microRNAs in lungs of aging Zmpste24 deficient mice

**Figure 7:**
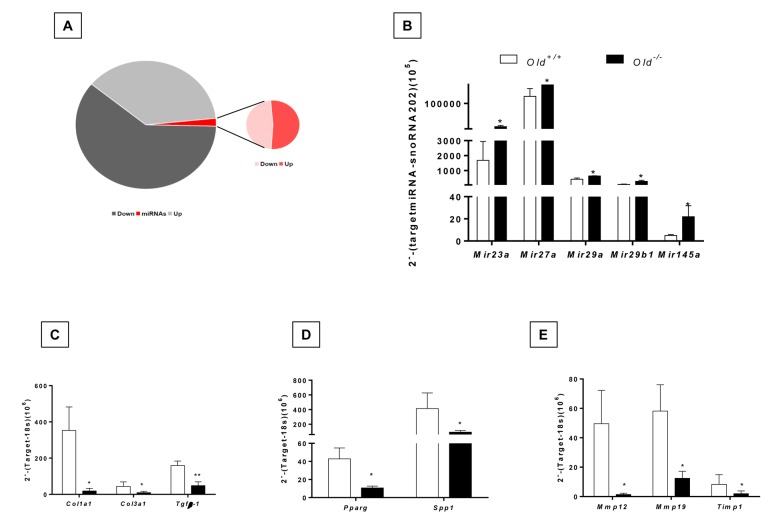
**Dysregulated miRNA expression and gene targets of bleomycin-treated lungs of old Zmpste24 deficient mice (n=3) compared to old WT (n=3) analyzed by quantitative RT-PCR.** (**A**) Pie graph showing significantly different mRNA (up= 627, down= 538) and miRNAs (up= 22, down= 20) in lungs of Zmpste24 deficient mice compared with their WT counterparts after bleomycin damage. (**B**) Expression levels of selected miRNAs. Gene expression of (**C**) miR29 targets, (**D**) miR27a targets, (**E**) mir145a targets. White bars represent mean expression in WT lungs, and black bars represent mean expression in Zmpste24 deficient mice ± S.E.M. *p < 0.05 and ** *p*< 0.01.

In the global RNA expression, we noticed that 42 microRNAs were differentially expressed, 22 upregulated and 20 down-regulated in the lungs from bleomycin injured *Zmpste24* deficient mice compared with their WT counterpart (Figure 7A, Supplementary Table 5). Several of these microRNAs were of interest because their targets are related with the extracellular matrix such as *miR23a*, *miR27a*, *miR29a, miR29b-1, miR491,* and *miR145a*, which were validated by qPCR. These five miRNAs were increased during the fibrotic response in old *Zmpste24* deficient mice (Figure 7B). The three members of the miR29 family (miR29a, miR29b-1, and miR29c) have been shown to target mRNAs of extracellular matrix proteins [16,17]. We found a downregulation of several miR29 targets in lungs from old *Zmpste24* deficient mice such as *Col1a1, Col3a1, and Tgfb-1*, which were corroborated by qPCR (Figure 7C). As mentioned, miR27a was upregulated in the lungs of old *Zmpste24* deficient mice after bleomycin injury. To investigate the role of this microRNA, we searched for targets in *silico* databases [18]. Matching with our results, we found two genes, *Spp1,* and *Ppar-γ.* That was downregulated in the lungs of old *Zmpste24* deficient mice compared with their WT counterpart after bleomycin injury. These results were validated by qPCR (Figure 7D).

miR145a that was also overexpressed in the lungs of Zmpste24 deficient mice targets among others, *Mmp12*, *Mmp13* and *Timp1* genes. As shown in Figure 7E, these genes had a lower expression in old bleomycin injured *Zmpste24* deficient mice as validated by qPCR.

## DISCUSSION

Aging is not only a major risk factor for the development of IPF but also worsens the fibrotic response and outcome in human and experimental fibrotic lung diseases [9,10,19]. Moreover, it has been shown that old mice display impaired resolution after lung injury [11]. Thus, aged mice may reflect more appropriately the physiopathological behavior of aging-associated human pulmonary fibrosis. However, there are considerable practical difficulties related to the generation of aged mice and most of the research in this field has been performed in mice 6-8 weeks old.

In this context, senescence-prone mice that exhibit accelerated aging, and genetically modified mice that result in age-related lung fibrosis have been explored as models that may resemble what occurs in IPF and other human fibrotic lung disorders [2,13,20].

In this study, we used the Zmpste24 deficient mice that display several features of premature aging [14]. We selected the time point of biological age in the rapid aging model to compare with old WT mice, using the strong evidence that Zmpste24 deficient mice had a remarkably aging phenotype since they are12 weeks old, including short lifespan (20 weeks old) [14,21–23]. For this reason, we selected as young, mice of 4 weeks age, and old, those of 13 weeks age to start observations [14]. Of note, no lung morphological differences were found between WT and Zmpste24 deficient mice by conventional light microscopy (not shown). Additionally, we analyzed whether aging affected the lung levels of Zmpste24. We found that the enzyme increase in old mice, and is mainly located in bronchioalveolar epithelial cells (Supplementary Figure 2).

We first compared the transcriptome signature of young and old WT and young and old *Zmpste24* deficient mice, and a number of differences showed-up. Interestingly for our study, in the WT mice, aging was associated with the upregulation of several extracellular matrix proteins (core matrisome) and of matrix metalloproteinases (matrisome-associated genes) [24]. The opposite, with the exception of *Spp1*, was observed in the *Zmpste24* deficient mice. Interestingly, although fibrillar collagen genes were not up upregulated, when we examined the lung hydroxyproline content we found a marked but similar increase of collagen with age suggesting that it represents an accumulation of the protein with aging. The fibrils were localized mainly adjacent to the airways.

Afterward, we evaluated the effect of age on the fibrotic response to bleomycin comparing the extent of the lesions and collagen deposition in young and old WT versus young and old *Zmpste24* deficient mice. The mouse model of bleomycin-induced lung injury is the most widely used for the study of lung fibrosis [25].

Strikingly, we found that while young WT and young *Zmpste24* deficient mice develop a similar architectural lung remodeling and increased collagen accumulation, the fibrotic lesions were remarkably attenuated in accelerated aged old *Zmpste24* deficient mice compared with physiologically aged WT.

To better understand the mechanisms by which old *Zmpste24* deficient mice are protected from bleomycin-induced lung fibrosis, we examined the transcriptome expression profiling of the aged mice. Our results demonstrated that several Core matrisome (extracellular matrix) genes and MMPs (Matrisome-associated genes) are downregulated in *Zmpste24* deficient mice.

Interestingly, one of them was osteopontin a strong profibrotic mediator [26]. Osteopontin increases fibroblast migration and proliferation, and induces the upregulation of type I collagen, and the down-regulation of MMP-1 expression. Moreover, osteopontin-deficient mice show a marked reduced collagen accumulation in response to bleomycin challenge compared with WT mice [27].

Interestingly, several transcription factors were over-expressed including paternally expressed 3 (Peg3) a mediator of p53 in response to DNA damage [28], the negative regulator of the inflammatory response nuclear receptor subfamily 4 group A member 1 (NR4A1), which regulates cytokine signaling and attenuates inflammation in lung epithelial cells [29], and RAR-related orphan receptor C (RORC), which regulates T-cell polarity and cytokine production [30]. Also, the transcription factor Krupple-like factor 15 (KLF15) involved in defense response and hypoxia inducible factor 3a (Hif3a) were also overexpressed [31]. Likewise, the mechanosensitive gene of early growth factor response 1 (Egr1) was also upregulated. This transcription factor was found overexpressed in mice protected from lung hyperventilation injury [32], and has an important role in the inflammatory response [33]. Accordingly, functional analysis revealed altered immune and inflammatory response.

Since considerable evidence indicates that miRNAs play critical roles in the pathogenesis of lung fibrosis either enhancing or attenuating fibrogenic pathways, we also examined the miRNA expression profile, and we identified a panel of 42 miRNAs that were differentially expressed. Among them, miR23a, miR27a, miR29a, miR29b-1, and miR145a, that target different components of extracellular matrix and can be considered anti-fibrotic, were increased in old *Zmpste24* deficient mice compared with old WT after bleomycin-injury.

One of them, miR-29b, inhibits TGF-β1, CTGF, and Smad3 signaling, acting as a counter-regulator of the strong profibrotic TGF-β/Smad3/CTGF axis [34].

Moreover, evaluation of the expression of miR-29a/b in lungs collected at different time points after bleomycin treatment revealed a gradual decrease during the development of fibrosis with a subsequent increase that correlates with the remission of the fibrotic lesions [16].

miR27-a, another upregulated miRNA in bleomycin-injured *Zmpste24* deficient mice, functions via negative-feedback mechanisms decreasing lung myofibroblast differentiation, and therapeutically mitigates bleomycin-induced lung fibrosis in mice [35]. Furthermore, *in silico* analysis predicts that one of its targets is osteopontin.

miRNA-491 has been evaluated mostly in cancer, but it has been shown that target mRNAs involved in strong profibrotic pathways including TGF-β/SMAD3/NF-κB and Wnt3a/β-catenin signaling pathways [36,37].

Certainly, several other mechanisms may contribute to the decreased fibrotic response of the *Zmpste24*-deficient mice to lung injury. For example, autophagy that results in the accumulation of damaged macromolecules, decreases during aging and is even exaggeratedly reduced in lung fibrosis. In sharp contrast, Zmpste24-deficient mice exhibit a pronounced activation of autophagic proteolysis caused by reduced activity of mTOR [38].

In conclusion, our results indicate that the absence of *Zmpste24* in aging mice results in impaired lung fibrotic response after injury which is likely associated to the dysregulation of fibrosis-related miRNAs.

## MATERIALS AND METHODS

### Animals

Young (4 weeks) and old (79 weeks) C57BL/6 wild type mice (WT) and young (4 weeks) and old (13 weeks) *Zmpste24* deficient mice were housed in specific pathogen-free conditions and used for different experiments. Mice genotypes were determined by PCR analysis from mouse tail DNA as previous described [14]. The Ethics Committee of the National Institute of Respiratory Diseases of Mexico (INER) approved all experiments.

### Bleomycin-induced lung fibrosis

Pulmonary fibrosis was induced by oropharyngeal administration of a single dose of 0.1U Bleomycin/g mice weight (BLEOLEM, Lemery) in a volume of 50µl saline solution. Control groups received only the vehicle. All mice were sacrificed at 21 days after bleomycin or saline administration (*Zmpste24-deficient mice*: 16 months and WT: 82 months). Lungs were perfused with sterile saline from right to left ventricle of the heart. Lungs were removed for fixation overnight in paraformaldehyde (right lung) or snap frozen in liquid nitrogen (left lung) followed by storage at –80°C.

### Hydroxyproline assay

Left lungs were dried at 110°C during 48 h and then hydrolyzed in 6 N HCl for 24 h. Samples of 5 μL were assayed as previously described [39,40]. Each sample was tested in duplicate and results were expressed as μg of hydroxyproline/mg of the dry left lung.

### Morphometric analysis

Right lungs were fixed by inflation using 4% paraformaldehyde at a continuous pressure of 25 cm H2O, and embedded in paraffin. Lung sections were either stained with hematoxylin-eosin or Masson trichrome and scored blindly for severity and extent of lung lesions. The severity of lung fibrosis was determined using a semiquantitative histopathological scoring method [40].

### RNA extraction and preparation

RNA was extracted using TRIzol reagent (Life Technologies, Grand Island, New York, NY) following the manufacturers' instructions, and purity and efficiency were verified by spectrophotometry (NanoDrop; Wilmington, DE) and bioanalysis (Agilent; Palo Alto, CA).

### Quantitative real-time PCR

One μg of RNA was treated with 1 unit of DNase and reverse transcribed into cDNA (RT2 First strand kit, Qiagen) according to the manufacturer's instructions. qPCR amplification was performed with specific FAM or VIC dye-labeled TaqMan probes for *Col1a1* (Mm00801666_g1), *Col3a1* (Mm00802300_m1), *Mmp8* (Mm00439509_m1)*, Mmp12* (Mm00500554_m1), *Mmp13* (Mm00439491_m1), *Mmp19* (Mm00491296_m1), Pparᵧ (Mm00440940_m1), *Spp1* (Mm00436767_m1), *Egr1* (Mm00656724_m1), *Eln* (Mm00514670_m1), *Tgfβ-1* (Mm01178820_m1), *Tgfβi* (Mm01337605_m1), *Timp1* (Mm00441818_m1) and normalized to *18S rRNA* expression (PE Applied Biosystems). Time PCR amplification was performed using BIORAD CFX-96 Real-Time PCR system (Bio-Rad) [41].

### Microarray analysis

The biotin-labeled cRNA was purified, fragmented, and hybridized to GeneChip™ Mouse Gene 2.0 ST Array (Affymetrix®). For each group, 100 ng of RNA from three different biological samples were used. The results were analyzed by R software (http://www.r-project.org/) [42] and Bioconductor (http://www.bioconductor.org/) [43]. To identify significant differences between gene expression in each condition, all data were analyzed by Limma linear model based on Bayes empirical method [44]. Representative data were considered significantly with higher *p*-values (adjusted *p*-value < 0.05).

The microarray data were submitted to the Gene Expression Omnibus https://www.ncbi.nlm.nih.gov/geo/ (access number GSE123293).

### Gene Ontology (GO) and Kyoto Encyclopedia of Genes and Genomes (KEGG Pathway analyses

For functional analysis, we used Gene Ontology enrichment tool at the Enrichr website. GO enrichment analysis is a computational method for inferring knowledge about an input gene set by comparing it to annotated gene sets representing prior biological knowledge. The data was graphed as Combined Score, a combination of the *p*-value and *z-*score calculated by multiplying the two scores as follows: *c* = ln(*p*) * z. Where c is the combined score, *p* is the *p*-value computed using Fisher's exact test, and *z* is the *z*-score computed to assess the deviation from the expected rank. The Combined Score provides a compromise between both methods and in several benchmarks and it has been shown that reports the best rankings when compared with other scoring schemes. KEGG database was used for pathway analysis of differential expression genes using gProfile software. For all comparison we considered differentially expressed genes with an adjusted *p-*value < 0.05 [45–47].

### microRNA expression

Total RNA was reverse transcribed using TaqMan miRNA reverse transcription kit (Applied Biosystems) following the manufacturer’s instructions, and amplification was performed using BIORAD CFX-96 Real-Time PCR system (Bio-Rad). MicroRNA expression was evaluated using following TaqMan probes mmu-miR23a-5p (002439), mmu-miR27a-3p (000408), mmu-miR29a-3p (002112), mmu-miR29b-3p (000413), mmu-miR145a-3p (002514). Expression of snoRNA202 (001232/AF357327) was used as an internal control. All experiments were performed with Taqman Gene Expression Assays (Applied Biosystems).

### Statistical analysis

Statistical differences were determined by two-way ANOVA followed by Tuckey test for quantitative PCR and hydroxyproline measurement. For two groups, differences were analyzed by Student’s *t*-test. Values of *p* < 0.05 were considered statistically signiﬁcant. Fibrosis score was evaluated by the nonparametric Kruskal-Wallis test followed by nonparametric Mann-Whitney U-test. Results are expressed as mean ± SD. or S.E.M., *p-*value < 0.05 was considered statistically significant. For gene tables, we only show identified genes in the Affymetrix annotation database. All graphs were made using Graphpad Prism Software Version 4.0 (Graphpad Software Inc., San Diego CA).

## Supplementary Material

Supplementary Figures

Supplementary Table 1

Supplementary Table 2

Supplementary Table 3

Supplementary Table 4

Supplementary Table 5
