# Supplementary material for: Accelerated aging induced by deficiency of Zmpste24 protects old mice to develop bleomycin-induced pulmonary fibrosis
Source: Aging (Albany NY). 2018 Dec 10;10(12):3881–96. doi: 10.18632/aging.101679 (PMC6326652; doi:10.18632/aging.101679)
Supplement: Supplementary Figures [file aging-10-101679-s001.pdf]

SUPPLEMENTARY FIGURES

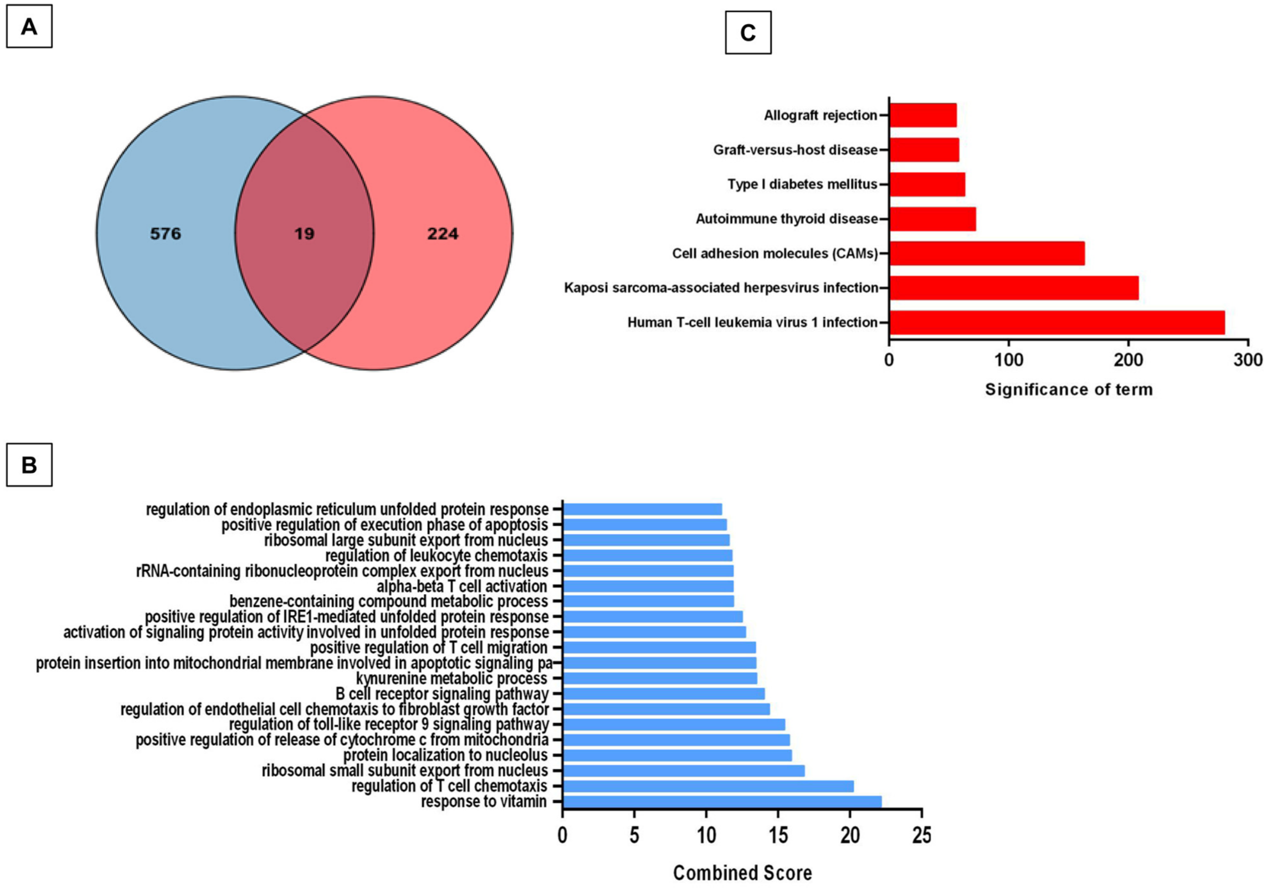

Supplementary Figure 1. Bioinformatic analysis of changes associated to accelerated lung aging compared to normal lung aging. (**A**) Venn diagram of dysregulated genes in natural aging (old WT vs young WT) in blue circle, and dysregulated genes in accelerated aging (old Zmpste24-/- vs young Zmpste24 -/-) in red circle. Light purple circle shows overlapping genes between both groups. (**B** and **C**) Gene ontology (**B**) and KEGG (**C**) functional analysis. Threshold criteria considered for the analysis are log fold-change > 1 or <-1 and p-value < 0.05 for genes, and >0.5 or z-0.5 for miRnas.

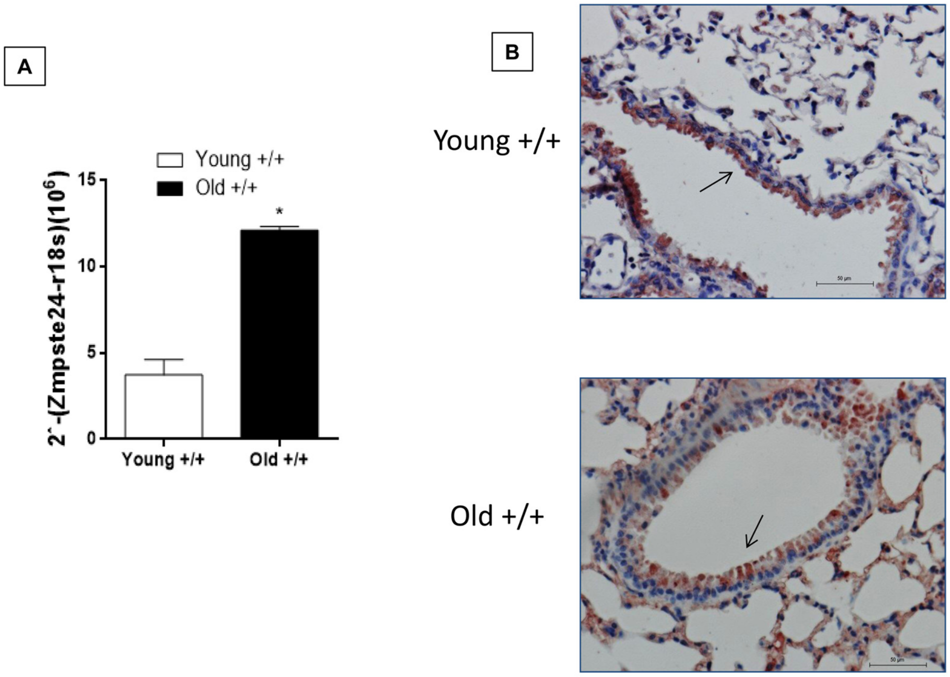

Supplementary Figure 2. Lung expression and localization of *Zmpste24* in old and young WT mice. (**A**) qPCR of Zmpste24 in lungs of old and young WT mice under basal conditions. Results are shown as mean ± SD. Statistical significance was determined by Student´s t-test (*$p < 0.05$). (**B**) Representative photomicrographs of immunohistochemical staining performed with specific primary antibody against Zmpste24 in lungs from old and young WT mice under basal conditions. Positive signal is observed in broncho-alveolar epithelial cells (black arrows). All sections were counterstained with hematoxylin. Scale bar: 50μm.
